# Coexistence in two-species competition with delayed maturation

**DOI:** 10.1007/s00285-023-02031-2

**Published:** 2023-12-19

**Authors:** Maud El-Hachem, Nicholas J. Beeton

**Affiliations:** Data61, CSIRO, Battery Point, Tasmania Australia

**Keywords:** Lotka–Volterra competition, Maturation, Distributed delay, Truncated Erlang distribution, Linear chain approximation, 34K20 Stability theory of functional-differential equations, 92D25 Population dynamics (general)

## Abstract

Inter- and intraspecific competition is most important during the immature life stage for many species of interest, such as multiple coexisting mosquito species that act as vectors of diseases. Mortality caused by competition that occurs during maturation is explicitly modelled in some alternative formulations of the Lotka–Volterra competition model. We generalise this approach by using a distributed delay for maturation time. The kernel of the distributed delay is represented by a truncated Erlang distribution. The shape and rate of the distribution, as well as the position of the truncation, are found to determine the solution at equilibrium. The resulting system of delay differential equations is transformed into a system of ordinary differential equations using the linear chain approximation. Numerical solutions are provided to demonstrate cases where competitive exclusion and coexistence occur. Stability conditions are determined using the nullclines method and local stability analysis. The introduction of a distributed delay promotes coexistence and survival of the species compared to the limiting case of a discrete delay, potentially affecting management of relevant pests and threatened species.

## Introduction

Population control technologies aim to replace or to suppress vectors of diseases, such as arthropods that carry and transmit pathogens to animals or humans. Risk assessment of the large-scale implementation of population control technologies relies in part on mathematical modelling of the competitive dynamics between the species to be replaced or eliminated and the species introduced, such as transgenic constructs (Beeton et al. 2020, 2022; Pagendam et al. 2020; Taghikhani et al. 2020). We are interested in competition between disease vectors such as mosquitoes, where maturation of the offspring is long compared to the lifespan of the adult. In this case, immature stages, hatching eggs and larvae foraging in a water reservoir are subject to far more intense competition for resources than adult mosquitoes, who have an abundant source of food (Noden et al. 2016).

Multi-species models of competition studied in the literature are often extensions of the continuous Lotka–Volterra competition model (Gause and Witt 1935; Hofbauer et al. 1987; Turchin 2013) or its discrete equivalent, the Leslie–Gower model (Leslie and Gower 1958). The Lotka–Volterra competition model incorporates density dependent growth that takes into account both intraspecific competition between individuals of the same species and interspecific competition between individuals of different species. Generalisations of the logistic Lotka–Volterra competition model, since the first successful applications to yeast and protozoans (Gause and Witt 1935), include non-linear competitive interactions (Gilpin and Ayala 1973) to capture growth behaviour for a wider diversity of species. Mechanistic approaches incorporate components of the processes that result in competition (Schoener 1976). For example, Beeton et al. (2020) included the process of choosing a mate when hybridising subspecies are competing.

Sexually mature individuals can be separated from immature individuals by using maturation delay, either in a discrete form assuming a single known delay period, or a distributed form containing a range of different delays in a known distribution. The end of the maturation period signifies that the individual is now able to breed. Modelling a maturation delay provides a simpler alternative to including age-structured subpopulations that require an extensive number of parameters to characterise each subpopulation. The kernel of a gamma distribution for a distributed delay stands as a more realistic option than a discrete delay (Blythe et al. 1984), allowing for more heterogeneity in representing the maturation by accommodating individual variation. Variability of the maturation duration could be a successful strategy for a species in response to a changing environment.

The insertion of a delay into the Lotka–Volterra competition model has been achieved by extending the delayed logistic model, called the Hutchinson–Wright model, to multiple species (Gopalsamy 1980; Gopalsamy and Aggarwala 1980; Smith 1995; Chen et al. 2010). The delay in the Hutchinson–Wright model causes growing, steady or declining oscillations around the equilibrium point (Murray 2002; Berezansky and Braverman 2003). Using the Hutchinson–Wright model as a template for adding a delay to the Lotka–Volterra competition leads to the existence of oscillations under some conditions. Biological populations are not easily described by oscillatory solutions, especially given sparse biological data. The oscillations observed in nature may not be directly related to maturation delay but may be due to external variations such as seasonal changes.

Here, we build on an alternative approach, proposed by Arino et al. (2006), that elaborates the model with a delay from a mechanistic point of view. This alternative to the delayed logistic model (Arino et al. 2006; Lin et al. 2018; Baker and Röst 2020) is obtained by replacing the birth term in the logistic growth by a function that depends on maturation delay. Arino et al. assume that the recruitment rate of immatures to the adult population is based on new births from the adult population *N* alive at past time $t-\tau$, i.e. $N(t-\tau )$, and on survival until the current time *t*. Death and intraspecific competition rates are instantaneous, and depend only on the current population. The number of remaining individuals that survive attrition during maturation is obtained by solving the equation without a birth term, $\textrm{d}N(s)/\textrm{d}s = -mN(s) - a N^2(s)$ at time *t*, using the known initial condition $N(t-\tau )$. The full model in Arino et al. (2006) takes the following form, including the resulting birth term,

1 $$\begin{aligned} \dfrac{\textrm{d}N}{\textrm{d}t} = r\dfrac{m N(t-\tau )}{m \exp {(m \tau )}+a (\exp {(m \tau )}-1)N(t-\tau )}-m N(t) - a N^2(t). \end{aligned}$$

The positive equilibrium, named the “delayed carrying capacity”, exists only if the maturation delay $\tau$ of the species is below a critical threshold, implying that the population goes extinct if the delay is too long. This approach has also been applied to the Lotka–Volterra competition model, with a discrete delay (Lin et al. 2022), to show that evolution favours a short maturation delay in order to increase the carrying capacity of the population.

In this work, we build on the delayed Lotka–Volterra competition model for two species by representing the maturation delay with a truncated Erlang distribution. Including a truncation point allows for the case where a juvenile must either mature within a defined period or die. We consider a population of adults colonising a new environment, like the introduction of flying insects into a cage, or the beginning of the rainy season for mosquitoes in a seasonal environment. In our approach, mature and immature individuals together form a population at equilibrium in an environment where a perturbation is introduced by inserting or removing adults of one or two species. In the next section, we derive the equations of the model with a distributed delay. In Sect. 3, we discuss the numerical results obtained, and we study the stability of the system. We complete the presentation of the results by discussing a biological example.

## A model with a distributed maturation delay

The Lotka–Volterra competition model for two species (Gause and Witt 1935) consists of two coupled differential equations

2 $$\begin{aligned} \dfrac{\textrm{d}\hat{N}_i}{\textrm{d}\hat{t}} = \hat{r}_i\hat{N}_i \left( 1-\dfrac{\hat{a}_i \hat{N}_i+\hat{b}_j \hat{N}_j}{\hat{K}_i}\right) - \hat{m}_i\hat{N}_i, \quad (i,j) = \{(1,2), (2,1)\}, \end{aligned}$$

where $\hat{N}_i(\hat{t})$ represents the abundance of species *i* as a function of time $\hat{t}$. All parameters are positive: the mortality rate $\hat{m}_i$, the reproduction rate $\hat{r}_i$, the carrying capacity $\hat{K}_i$ of the breeding environment, the density dependence effect $\hat{a}_i$ of species *i* on its conspecifics, and the effect $\hat{b}_j$ of species *j* on species *i*. It is assumed that $\hat{r}_i > \hat{m_i}$. The non-trivial equilibrium for the single species model when $\hat{b}_j= 0$ is $\hat{K}_i(\hat{r}_i-\hat{m}_i)/(\hat{r}_i\hat{a}_i)$. The model in (2) is often studied under two types of competition, or “struggle for existence” (Gause and Witt 1935). The case of weak interspecific competition is characterised by the condition

3 $$\begin{aligned} \hat{a}_1\hat{a}_2>\hat{b}_1\hat{b}_2, \end{aligned}$$

where the two species compete for common food or resources while also belonging to different ecological niches, such that one species has particular resources that are not consumed by the other species. Weak interspecific competition can in some cases give rise to coexistence where both species survive. Strong interspecific competition is defined by the complement of (3) such that

4 $$\begin{aligned} \hat{a}_1\hat{a}_2\le \hat{b}_1\hat{b}_2, \end{aligned}$$

and represents a situation where two species compete strongly for common resources, leading to the less numerous species losing the competition and being excluded.

We introduce the normalised variables $N_i = \hat{N}_i/\hat{K}_i$, $b_i=\hat{b}_i\hat{K}_i/\hat{K}_j$, $a_i = \hat{a}_i$ and non-dimensionalise (2)

5 $$\begin{aligned} \dfrac{\textrm{d}N_i}{\textrm{d}t} = r_i N_i\left( 1-a_i N_i-b_j N_j\right) - m_i N_i, \qquad (i,j) = \{(1,2), (2,1)\}, \end{aligned}$$

where $$t=\hat{t}$$, $$r_i=\hat{r}_i$$ and $$m_i=\hat{m}_i$$.

We now introduce maturation delays $s_i$ for $i=\{1,2\}$. We assume that the population size of immature offspring decreases due to inter- and intraspecific competition. We also assume that adults experience density independent mortality and negligible competition. We reformulate the Lotka–Volterra competition model for two species in (5) by generalising the expression of the delayed growth for one species calculated in (1), such that

6 $$\begin{aligned} \dfrac{\textrm{d}N_i(t)}{\textrm{d}t} = r_i {\mathcal {R}}_i(t) - m_i N_i(t) , \end{aligned}$$

where the expression $r_i {\mathcal {R}}_i(t)$ is the number of immatures born (e.g. eggs) at time $t-s_i$ that survive to maturation and emerge as adults at time *t*. The birth rate of new immatures at time $t-s_i$ is assumed to be proportional to the size of the adult population at time $t-s_i$, where $r_i$ is the number of hatching offspring per adult, such that ${\mathcal {R}}_i(t-s_i) =N_i(t-s_i)$. Density dependent mortality then applies to these offspring from time $t - s_i$ until time *t*. We find ${\mathcal {R}}_i(t)$ by evaluating

7 $$\begin{aligned} \int _{t-s_i}^{t}\dfrac{1}{{\mathcal {R}}_i}\textrm{d}{\mathcal {R}}_i = \int _{t-s_i}^{t}\left[ -\mu _i - a_i N_i(s) - b_{j} N_j(s) \right] \textrm{d}s, \end{aligned}$$

where $1/{\mathcal {R}}_i \ \textrm{d}{\mathcal {R}}_i$ is the per capita rate of change of the immature population born at time $t-s_i$ and expected to emerge at *t*, where $\mu _i>0$ is the rate of mortality of the immature offspring, and $a_i N_i(s) + b_j N_j(s)$ is the density dependent mortality specified in Eq. (5). The left hand side of Eq. (7) becomes $\log ({\mathcal {R}}_i(t)) -\log ({\mathcal {R}}_i(t-s_i)) =\log ({\mathcal {R}}_i(t)) -\log (N_i(t-s_i))$ and we then obtain

8 $$\begin{aligned} {\mathcal {R}}_i(t)=N_i(t-s_i) \exp {\left( -s_i \mu _i-\int _{t-s_i}^{t} \left[ a_i N_i(s) +b_j N_j(s) \right] \textrm{d}s\right) }. \end{aligned}$$

We also define the function $f_i(t)$ as

9 $$\begin{aligned} f_i(t) = \frac{1}{s_i} \int _{t-s_i}^t N_i(s) \textrm{d}s. \end{aligned}$$

We can then substitute Eq. (8) into Eq. (6), and use Eq. (9) to define a coupled ODE model for two species with a discrete delay, such that

10 $$\begin{aligned} \dfrac{\textrm{d}N_i}{\textrm{d}t}&=r_i N_i\left( t-s_i\right) \exp {\big (-\mu _i s_i -s_i\left[ a_i f_i(t)+b_j f_j(t) \right] \big )}-m_i N_i(t), \end{aligned}$$

11 $$\begin{aligned} \dfrac{\textrm{d}f_i}{\textrm{d}t}&= \dfrac{N_i(t) - N_i \left( t-s_i\right) }{s_i}, \end{aligned}$$

where $(i,j) = \{(1,2),(2,1)\}$. The function $f_i(t)$ represents the mean population density during maturation and $\textrm{d}f_i/ \textrm{d}t$ when negative corresponds to the mean population loss during maturation due to intra- and inter-specific competition across the maturation period. The initial conditions $N_i(t)=\phi _i(t)>0$ for $t\le 0$, are bounded continuous functions mapping from $(-\infty ,0]$ to ${{\mathbb {R}}^+}$. The initial conditions, called history, describe a scenario where the population of adults at time $t=0$ is positive and where the configuration of the population at $t<0$ is at equilibrium, including the trivial equilibrium where $N_i(t<0)=0$. A perturbation occurs at $t=0$ when the population of adults increases or decreases.

We aim to convert the discrete delay described in Eq. (11) into a distributed delay, where maturation time varies randomly between individuals according to a defined probability distribution. The kernel used is the probability density function (PDF) of the gamma distribution

12 $$\begin{aligned} G\left( s\mid \beta ,p\right) = \dfrac{s^{p-1} {\beta }^p \exp {\left( -\beta s\right) }}{\Gamma (p)} \end{aligned}$$

where $p\ge 0$ is the shape parameter of the distribution, $\beta >0$ is the rate parameter of the distribution and $\Gamma (p)$ is the gamma function. The area under the curve in (12) is $\int _{0}^{\infty } G\left( s\mid \beta ,p\right) \textrm{d}s=1$ and the average delay is $\int _{0}^{\infty } s G\left( s\mid \beta ,p\right) \textrm{d}s=p/\beta$. The limit of the probability density function in (12) when $p\rightarrow \infty$ is a shifted Dirac distribution, $\delta (s-p/\beta )$, corresponding to the kernel of the discrete delay (Smith 2011).

It is more relevant computationally to use a bounded support, consistent with a finite interval of integration. A limited support of the distributed kernel signifies biologically that there is an upper limit to the amount of time necessary for an individual to mature and that no maturation is possible beyond this limit. The threshold for the maturation period is an intrinsic characteristic of the species. We use the reflected-shifted-truncated gamma distribution, as studied in Waymyers et al. (2018), to bound the support of the delay kernel. The reflected-shifted-truncated gamma distribution is defined by the probability density function

13 $$\begin{aligned} \hat{G}(u\mid \beta ,p,\Delta ) = \dfrac{u ^{\left( p-1\right) } {\beta }^p \exp {\left( -\beta u\right) }}{\Gamma (p)-\Gamma \left( p,\beta \Delta \right) }, \end{aligned}$$

where $\Delta >0$ is the shift and $u=\Delta -s$, $\Gamma \left( p, \beta \Delta \right)$ is the incomplete upper gamma function, such that $\Gamma \left( x,a\right) = \int _{a}^{\infty } t^{x-1}\exp {(-t)}\,\textrm{d}t$ for any real $x > 0$. The PDF in (13) is normalised so that similarly the area under the curve $\int _{0}^{\infty } \hat{G}\left( u\mid \beta ,p,\Delta \right) \textrm{d}s=1$. The mean of (13) is $\Delta -\beta [\Gamma (p+1)-\Gamma (p+1,\beta \Delta )]/[\Gamma (p) -\Gamma (p,\beta \Delta )]$. The truncated distribution used for a kernel with bounded support appears also in Vittadello et al. (2021) to describe the distributed delay in a heterogeneous biological population of cells.

**Fig. 1:**
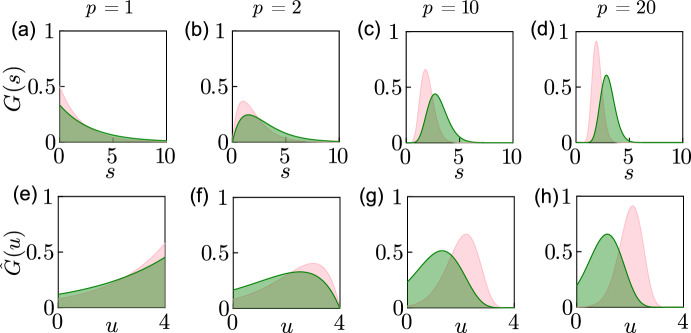
Probability density function of the gamma distribution for different *p* and $\beta$. The probability density function $G(s\mid \beta ,p)$ from (12) is shown for $p=1,2,10$, and 20 in (**a**), (**b**), (**c**) and (**d**) respectively, where $\beta =p/2$ in the pink shape and $\beta =p/3$ in the green shape. The reflected shifted truncated probability density function $\hat{G}(u\mid \beta ,p,\Delta )$ (13) is shown for $\Delta = 4$ in (**e**), (**f**), (**g**) and (**h**) corresponding to the parameters *p* and $\beta$ of (**a**), (**b**), (**c**) and (**d**) respectively (colour figure online)

Figure 1 shows the skewness of the probability density function in (12) for different values of *p*, represented by a pink shape for a mean delay $p/\beta =2$ and by a green shape for a mean delay $p/\beta =3$. The peak or mode of the distribution is at the location $s=0$ when $p=1$ (Fig. 1a). The mode moves away from $s=0$ towards the position $p/ \beta$ when $p=2, 10$ and 20 (Fig. 1b–d). The probability density functions in Fig. 1e–h are obtained from the PDFs in (a)–(d) after a reflection over the y-axis, followed by a shift of $\Delta = 4$ to the right and a left truncation at $s=0$. Classical models using delays for biological populations favour shifted distributions (Blythe et al. 1984) as they assume a minimum mandatory time for maturation. The reflected-shifted-truncated gamma distribution can approximate the minimum mandatory time for maturation by using $p\gg 1$ as in Fig. 1d, h where the probability that the delay occurs between $u= \Delta$ and $u= \Delta - 1$ is nearly zero, corresponding to a minimum mandatory time of maturation equal to one.

The discrete delay described in Eq. (11) in the form $N(t-s_i)$ is equivalent to the integral $\int ^\infty _0 N\left( t-s\right) \delta \left( s-s_i\right) \textrm{d}s$ (Smith 2011) taken over all possible delays *s*, where $\delta$ is the Dirac function. We can use this formulation to convert our discrete delay into a distributed delay using the reflected-shifted-truncated gamma distribution in Eq. (13). The delay $N(t-s_i)$ can then be replaced by $\int ^\infty _0 N\left( t-u\right) \hat{G}\left( u\mid \beta ,p,\Delta \right) \textrm{d}u$, giving us the final set of model equations

14 $$\begin{aligned} \dfrac{\textrm{d}N_i}{\textrm{d}t}&= r_i \int _0^\infty N_i \left( t-u\right) \exp {\left( -\mu _i u - \Delta _i \left[ a_i f_i(t)+b_j f_j(t) \right] \right) } \hat{G}\left( u\mid \beta ,p,\Delta _i\right) \textrm{d}u \nonumber \\&\quad -m_i N_i(t), \end{aligned}$$

15 $$\begin{aligned} \dfrac{\textrm{d}f_i}{\textrm{d}t}&= \frac{1}{\Delta _i} \left( N_i(t) - \int ^\infty _0 N\left( t-u\right) \hat{G} \left( u\mid \beta ,p,\Delta _i\right) \textrm{d}u \right) . \end{aligned}$$

where the use of $s_i$ in the denominator of Eq. (11) is now replaced with a scaling factor $\Delta _i$. Note that the distributed delay has been applied independently to $N_i(t)$ and $f_i(t)$, so that density dependent mortality of immatures is independent of the maturation period *u*. Distributed delays are well studied in the literature; for example, the alternative logistic model (1) for one species is generalised with a distributed delay in Lin et al. (2018). However, our work is different from previous mechanistic models that consider a maturation delay. To our knowledge, this is the first analysis of a distributed delay model of two species where there is no competition among adults.

The linear chain approximation (MacDonald 1978; Smith 2011; Wolkowicz et al. 1997) allows us to transform a system of delay differential equations (DDEs) with a distributed delay represented by a gamma distribution into a system of ordinary differential equations. We define the term containing the distributed delay in the right hand side of $\textrm{d}N_i/\textrm{d}t$ as

16 $$\begin{aligned} \left( \dfrac{\beta _i}{\beta _i+\mu _i}\right) ^{p_i}\int _{0}^{\infty } N_i\left( t-u\right) \left[ \dfrac{\Gamma (p_i)}{\Gamma (p_i)-\Gamma \left( p_i,\beta _i\Delta _i\right) }\right] G \left( u\mid \beta _i,p_i,\mu _i\right) \textrm{d}u, \end{aligned}$$

where

17 $$\begin{aligned} G\left( u\mid \beta _i,p_i,\mu _i\right) = \dfrac{u^{(p_i-1)} {\left( \beta _i+\mu _i \right) }^{p_i} \exp {\left[ -\left( \beta _i+\mu _i \right) u\right] }}{\Gamma (p_i)}, \end{aligned}$$

for $u=\Delta _i -s$. The expression $\Gamma (p_i)/\left[ \Gamma (p_i)-\Gamma \left( p_i,\beta _i\Delta _i\right) \right]$ in (16) is the normalising constant that originates from the PDF of the truncated gamma distribution. The PDF $G\left( u\mid \beta _i,p_i,\mu _i\right)$ associated with the delay *u* must be multiplied by the normalising constant as the distribution is truncated.

We will then express the derivative of the PDF as a linear combination of PDFs. The kernel function in Eq. (17) satisfies the initial value problem

18 $$\begin{aligned} \dfrac{\textrm{d}G\left( u\mid \beta _i,1,\mu _i\right) }{\textrm{d}u}&=-\left( \beta _i + \mu _i\right) G \left( u\mid \beta _i,1,\mu _i\right) ,\nonumber \\ G\left( 0\mid \beta _i,1,\mu _i\right)&= \beta _i+\mu _i, \end{aligned}$$

19 $$\begin{aligned} \dfrac{\textrm{d}G\left( u\mid \beta _i,k,\mu _i\right) }{\textrm{d}u}&=\left( \beta _i + \mu _i\right) \left[ G\left( u\mid \beta _i,k-1,\mu _i\right) - G\left( u\mid \beta _i,k,\mu _i\right) \right] ,\nonumber \\&\quad G\left( 0\mid \beta _i,k,\mu _i\right) = 0, \end{aligned}$$

where $k=2 \ldots p_i$. We aim to work with a finite number of ordinary differential equations (ODEs), such that $p_i\in {\mathbb {N}}^+$ and $p_i\ge 1$. The results in this work are obtained by considering $G\left( u\mid \beta _i,p_i,\mu _i\right)$ as the Erlang distribution, a special case of the gamma distribution, where the shape of the distribution $p_i$ is discretised such that $\Gamma (p_i)=(p_i-1)!$ and $\Gamma \left( p_i, \beta _i\Delta _i\right) =(p_i-1)! \exp {(-\beta _i\Delta _i)}\sum _{k=0}^{p_i-1}(\beta _i\Delta _i)^k/k!$ for any integer $p_i\ge 1$. The Erlang distribution with a shape $p_i$ and a rate $\beta _i$ gives the elapsed time until a chain of $p_i$ independent events of maturation have occurred at a rate $\beta _i$.

The integral in Eq. (16) is defined as the function $x_{i,p_i}(t)$ in the system of equations

20 $$\begin{aligned} x_{i,k}(t)=\left[ \dfrac{\Gamma (p_i)}{\Gamma (p_i)-\Gamma \left( p_i,\beta _i\Delta _i\right) }\right] \int _{0}^{\infty } N_i\left( t-u\right) G\left( u\mid \beta _i,k,\mu _i\right) \textrm{d}u, \end{aligned}$$

where $k=1 \ldots p_i$ and $x_{i,k}(t)$ is a continuous bounded function mapping from $(-\infty ,0]$ to ${\mathbb {R}}$. The same process is applied to the term containing the distributed delay in the right hand side of $\textrm{d}f_i/\textrm{d}t$ that we define as the function $y_{i,p_i}(t)$ from the system of equations

21 $$\begin{aligned} y_{i,k}(t)=\left[ \dfrac{\Gamma (p_i)}{\Gamma (p_i)-\Gamma \left( p_i,\beta _i\Delta _i\right) }\right] \int _{0}^{\infty } N_i\left( t-u\right) G\left( u\mid \beta _i,k\right) \textrm{d}u, \end{aligned}$$

where $k=1 \ldots p_i$ and $y_{i,k}(t)$ is a continuous bounded function mapping from $(-\infty ,0]$ to ${\mathbb {R}}$. The system of ODEs made up of the derivatives of $x_{i,k}(t)$ with respect to *t* can be obtained using the Leibniz integral rule, by applying the properties of the derivative of the convolution and by using Eqs. (18)–(19). The same approach yields the system of ODEs constituting the derivatives of $y_{i,k}(t)$ with respect to *t*. We obtain the following system of $2(p_1+p_2+2)$ ordinary differential equations

22 $$\begin{aligned} \dfrac{\textrm{d}N_i}{\textrm{d}t}&= r_i \left( \dfrac{\beta _i}{\beta _i+\mu _i}\right) ^{p_i}x_{i,p_i} \exp {\left( -\Delta _i\left[ a_i f_i + b_j f_j\right] \right) } - m_i N_i, \end{aligned}$$

23 $$\begin{aligned} \dfrac{\textrm{d}x_{i,1}}{\textrm{d}t}&=\dfrac{\left( \mu _i + \beta _i\right) \left( N_i - x_{i,1}\right) }{1-\exp {(-\beta _i\Delta _i)} \textrm{e}_{p_i-1}(\beta _i\Delta _i)}, \end{aligned}$$

24 $$\begin{aligned} \dfrac{\textrm{d}x_{i,k}}{\textrm{d}t}&=\dfrac{\left( \mu _i + \beta _i\right) \left( x_{i,k-1} - x_{i,k}\right) }{1-\exp {(-\beta _i\Delta _i)} \textrm{e}_{p_i-1}(\beta _i\Delta _i)}, \end{aligned}$$

25 $$\begin{aligned} \dfrac{\textrm{d}f_i}{\textrm{d}t}&=\dfrac{N_i - y_{i,p_i} }{\Delta _i}, \end{aligned}$$

26 $$\begin{aligned} \dfrac{\textrm{d}y_{i,1}}{\textrm{d}t}&= \dfrac{\beta _i \left( N_i - y_{i,1}\right) }{1-\exp {(-\beta _i\Delta _i)}\textrm{e}_{p_i-1} (\beta _i\Delta _i)}, \end{aligned}$$

27 $$\begin{aligned} \dfrac{\textrm{d}y_{i,k}}{\textrm{d}t}&= \dfrac{\beta _i \left( y_{i,k-1} - y_{i,k}\right) }{1-\exp {(-\beta _i\Delta _i)} \textrm{e}_{p_i-1}(\beta _i\Delta _i)}, \end{aligned}$$

for $(i,j) = \{(1,2), (2,1)\}$, where $k=2 \ldots p_i$ and $\textrm{e}_{p_i-1}(\beta _i\Delta _i)=\sum _{l=0}^{p_i-1}(\beta _i\Delta _i)^l/l!$ is the exponential sum function.

## Results and discussion

In this section, we present the numerical solutions of the system of ODEs (22)–(27) and the conditions of existence of the equilibrium points. We combine the method of nullclines and some results from local stability analysis to understand the features of the system at equilibrium.

### Numerical solutions of the system of ODEs

The numerical solutions of the system of ODEs (22)–(27) are obtained using the Julia programming language; specifically with the solver BS3 in the package *DifferentialEquations.jl* (Rackauckas and Nie 2017). This solver is an implementation of the Bogacki-Shampine method (Bogacki and Shampine 1989), an explicit three-stage, third order Runge–Kutta method with an adaptative step size for the time domain. The tolerance is set to $1\times 10^{-6}$ to obtain the numerical results presented in this work. The code in Julia that solves the system of ODEs (22)–(27) is available on GitHub.

**Fig. 2:**
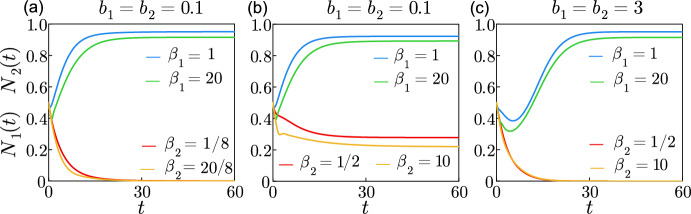
Numerical solutions illustrating survival, coexistence and competitive exclusion. The numerical solutions of the system of ODEs (22)–(27) are shown starting with initial conditions $N_1(0)=N_2(0)=0.5$ with $x_{i,k}(0)=y_{i,k}(0)=f_{i}(0)=0$. The parameters used are $m_1=m_2=\mu _1=\mu _2=0.3$, $r_1=r_2=1$, $a_1=a_2=1$, with $b_1=b_2=0.1$ in (**a**) and (**b**), and $b_1=b_2=3$ in (**c**), $p_1/\beta _1=1, p_2/\beta _2=8$ in (**a**), $p_1/\beta _1=1, p_2/\beta _2=2$ in (**b**) and (**c**). The shift used for the truncation is: **a** $\Delta _1=4.60$ and 1.59 when $p_1=1$ and 20 respectively and $\Delta _2=36.84$ and 12.74 when $p_2=1$ and 20 respectively, **b**–**c** $\Delta _1=4.60$ and 9.21 when $p_1=1$ and 20 respectively, and $\Delta _2=1.59$ and 3.19 when $p_2=1$ and 20 respectively. The solutions $N_1(t)$ and $N_2(t)$ are shown in blue and in red respectively when $p_1=p_2=1$, and in green and yellow respectively when $p_1=p_2=20$ (colour figure online)

Figure 2 shows numerical solutions $N_1(t)$ and $N_2(t)$ from (22)–(27) with parameters $m_1=m_2=\mu _1=\mu _2=0.3$, $r_1=r_2=1$ and $a_1=a_2=1$ constant accross all cases. The parameters $b_1$, $b_2$, $\beta _1$, $\beta _2$, $p_1$, $p_2$, $\Delta _1$ and $\Delta _2$ are chosen so that the resulting solutions illustrate extinction of one species in Fig. 2a, stable coexistence in Fig. 2b, and competitive exclusion in Fig. 2c. Note that these parameters chosen for Fig. 2 do not represent known biological species, though an example for two known species using biologically plausible parameters is discussed in Sect. 3.4. The truncation position is chosen to be at the 99th percentile of each maturation delay distribution (where the cumulative distribution function is equal to 0.99). The survival of species 1 in Fig. 2a is achieved by choosing $\beta _1=1$ when $p_1=1$ (blue line) and $\beta _1=20$ when $p_1=20$ (green line), and the extinction of species 2 is obtained by choosing $\beta _2=1/8$ when $p_2=1$ (red line) and $\beta _2=20/8$ when $p_2=20$ (yellow line). Comparison of Fig. 2a (where species 2 goes extinct) and Fig. 2b (where species 2 survives) shows that the rate $\beta _2$ must be increased from $\beta _2=1/8$ in Fig. 2a to $\beta _2=1/2$ in Fig. 2b when $p_2=1$, and from $\beta _2=20/8$ to $\beta _2=10$ when $p_2=20$, to ensure survival. The criterion for weak interspecific competition as defined in Eq. (3) is used in 2b, where the maturation period is short enough that the population gain due to birth is able to compensate for the loss due to mortality, resulting in stable coexistence of both species. Conversely, competitive exclusion in Fig. 2c is obtained by using the criterion for strong interspecific competition in Eq. (4). Species 1 wins the competition in Fig. 2c where the rate $\beta _1=1$ is greater than $\beta _2=1/2$ when $p_1=p_2=1$ and $\beta _1=20$ is superior to $\beta _2=10$ when $p_1=p_2=20$. We do not show the total extinction of both species that correspond to a maturation delay too long for each species to survive. The condition that relates the survival of each species to the distributed maturation delay is given in Sect. 3.2.1.

**Fig. 3:**
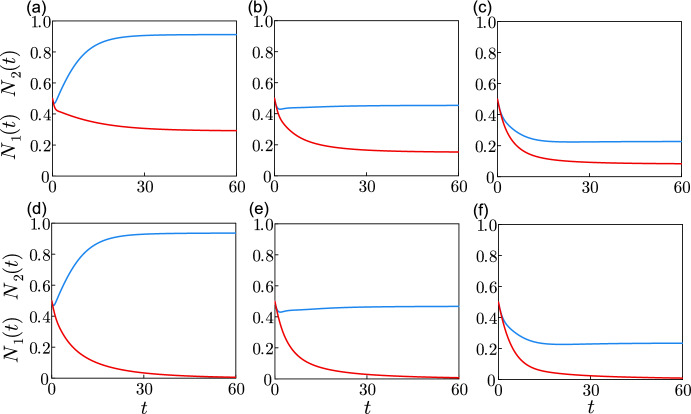
Numerical solutions at first and second quartiles, and at 99th percentile of the Chi-squared distribution. The solutions of system of ODEs (22)–(27) are shown starting with initial conditions $N_1(0)=N_2(0)=0.5$ and $x_{i,k}(0)=y_{i,k}(0)=f_{i}(0)=0$, for $m_1=m_2=\mu _1=\mu _2=0.2$, $r_1=r_2=1$, $a_1=a_2=1$, $b_1=b_2=0.1$, $\beta _1=\beta _2=1/2$, with $p_1=2$ and $p_2=3$ in (**a**)–(**c**), and $p_1=2$ and $p_2=5$ in (**d**)–(**f**). The solutions $N_1(t)$ and $N_2(t)$ are shown in blue and in red respectively, where $\Delta _1$ and $\Delta _2$ correspond to the first quartile in (**a**) and (**d**), to the second quartile in (**b**) and (**e**) and to the 99th percentile in (**c**) and (**f**) (colour figure online)

The Chi-squared distribution is a special case of the gamma distribution (12) with a rate of 1/2 and a shape of *k*/2, where *k* are the degrees of freedom, as illustrated in pink in Fig. 1a for a shape of one. Figure 3 presents the numerical solutions $N_1(t)$ and $N_2(t)$ from (22)–(27) where $\beta _1=\beta _2=1/2$ as in the Chi-squared distribution and $p_i=k/2$. We show how the solutions are different when the shifts of the truncation $\Delta _1$ and $\Delta _2$ correspond to the first quartile, the second quartile or the 99th percentile of the Chi-squared distribution, equivalent to the location where the cumulative distribution is 0.25, 0.50 and 0.99. We obtain coexistence for any quantile shown in (a)–(c) when $p_1=2$ and $p_2=3$. We obtain the survival of only species 1 when $p_1=2$ and $p_2=5$ for any quantile shown in (d)–(f). Figure 3 illustrates how the type of equilibrium does not change with the quartile used for the choice of $\Delta _1$ or $\Delta _2$. The magnitudes of $N_1$ and $N_2$ vary significantly with $\Delta _1$ and $\Delta _2$ when the truncation chosen corresponds to a percentile lower than the 99th percentile. The difference indicates that the truncation must be chosen carefully so it corresponds to a high percentile of the gamma distribution.

### Stability analysis

It can be easily shown that the solutions $N_1(t)$ and $N_2(t)$ are limited to the positive quadrant and bounded. By examining Eqs. (22)–(27), we can also readily show that any value of $f_i(t) = \bar{f}_i$ at equilibrium is proportional to $N_i(t) = \bar{N}_i$ at equilibrium such that $\bar{f}_i=p_i\displaystyle \big [1-\exp {(-\beta _i\Delta _i)}\,\textrm{e}_{p_i-1}(\beta _i\Delta _i)\big ]/(\beta _i\Delta _i)\ \bar{N}_i$, and that $\bar{x}_i=\bar{y}_i=\bar{N}_i$. We can then directly examine the equilibrium points of Eqs. (22)–(27) in the plane $(N_1,N_2)$. We can further show that the subsystem of Eqs. (22)–(24) being at equilibrium implies that the subsystem of Eqs. (25)–(27) is also at equilibrium, and vice-versa. As a result, only Eqs. (22)–(24) need be studied to determine the equilibrium states, and we thus consider that $f_i(t)$ and $y_{i,k}(t)$ are not relevant to describe the history in regards to equilibria. For numerical simulations, we choose initial conditions where either $f_i(0) = \bar{f}_i$ and $y_{i,k}(0) =\bar{y}_{i,k}$ or $f_i(0) =0$ and $y_{i,k}=0$, with $N_i(0) > 0$ and $x_{i,k}(0) \ge 0$.

#### Equilibrium points and nullclines

We present the existence conditions of the equilibrium points of Eqs. (22)–(27). Two outcomes can be distinguished visually in Figs. 2 and 3: the boundary equilibrium where one species is extinct while the other survives, and the coexistence equilibrium where both species coexist. The boundary equilibrium where $\bar{N}_i>0$ and $\bar{N}_j=0$, for $(i,j) = \{(1,2), (2,1)\}$, satisfies the condition

28 $$\begin{aligned} r_i\left( \dfrac{\beta _i}{\beta _i+\mu _i}\right) ^{p_i} \exp {\left( \dfrac{-a_ip_i[1-\exp {(-\beta _i\Delta _i)} \textrm{e}_{p_i-1}(\beta _i\Delta _i)]\bar{N}_i}{\beta _i}\right) } - m_i = 0, \end{aligned}$$

that corresponds to equilibrium points

29 $$\begin{aligned} \left( \bar{N}_i, \bar{N}_j\right) = \left( \dfrac{\beta _i \ln \left[ \dfrac{r_i}{m_i}\left( \dfrac{\beta _i}{\beta _i+\mu _i} \right) ^{p_i}\right] }{a _ip_i[1-\exp {(-\beta _i\Delta _i)} \textrm{e}_{p_i-1}(\beta _i\Delta _i)]} , 0\right) . \end{aligned}$$

We can use l’Hôpital’s rule to find that the limit of $-\ln \left( \beta _i/(\beta _i+\mu _i) \right) ^{ p_i}$ when $p_i\rightarrow \infty$ is $\mu _i(p_i/\beta _i)$ assuming that $p_i/\beta _i$ is a constant. The result is useful to understand what happens to the equilibrium point when the kernel of the distributed delay approaches the kernel of a discrete delay $\delta (s-p_i/\beta _i)$. The abundance at the semitrivial equilibrium (28) is such that $\bar{N}_i\rightarrow \infty$ when $p_i/\beta _i \ll 1$, exceeding $(r_i-m_i)/(r_i a_i)$, the expected carrying capacity for the single species model without a delay. The reason why the abundance in the delayed model exceeds that of the single species model with no delay is that density dependence and interspecific competition take effect in the space where the offspring are maturing, and are considered negligible in the space where the adults live. The offspring maturing in a short delay would very quickly vacate the space occupied, and would liberate resources for new offspring. A maturation delay approaching zero would thus mean a carrying capacity approaching infinity.

The condition of survival for species *i* in (29) corresponds to the critical condition

30 $$\begin{aligned} \ln (r_i) - \ln (m_i) + p_i \left[ \ln \beta _i-\ln (\beta _i+\mu _i)\right] > 0. \end{aligned}$$

Extinction occurring when condition (30) is false is not caused by competition. The species is simply extinct because the maturation period is too long for the species to survive at the current rates of reproduction and mortality.

We examine the following conditions for the existence of the equilibrium where both $\bar{N}_1$ and $\bar{N}_2$ are positive,

31 $$\begin{aligned}&r_1\left( \dfrac{\beta _1}{\beta _1+\mu _1}\right) ^{p_1} \exp {\left( -\dfrac{a_1p_1\bar{N}_1\alpha _1}{\beta _1} - \dfrac{b_2p_2\bar{N}_2\alpha _2}{\beta _2} \right) } - m_1 = 0, \end{aligned}$$

32 $$\begin{aligned}&r_2\left( \dfrac{\beta _2}{\beta _2+\mu _2}\right) ^{p_2} \exp {\left( - \dfrac{a_2p_2\bar{N}_2\alpha _2}{\beta _2} - \dfrac{b_1p_1\bar{N}_1\alpha _1}{\beta _1} \right) } - m_2 = 0, \end{aligned}$$

with

33 $$\begin{aligned} \alpha _i=[1-\exp {(-\beta _i\Delta _i)}\,\textrm{e}_{p_i-1} (\beta _i\Delta _i)], \quad i=1,2, \end{aligned}$$

where $\alpha _i$ is the inverse of the normalising constant of the PDF of the truncated Erlang distribution such that $\alpha _i\rightarrow 1$ when $\Delta _i\rightarrow \infty$. The equilibrium point $\left( \bar{N}_1, \bar{N}_2\right)$ following the conditions (31)–(32) correspond to

34 $$\begin{aligned} \left( \dfrac{\tau _1 b_2 A_2 -\tau _2 a_2 A_1}{ \tau _1\tau _2 (a_1 a_2-b_1 b_2)},\dfrac{\tau _2 b_1 A_1-\tau _1 a_1 A_2}{\tau _1 \tau _2 (a_1 a_2 -b_1 b_2 )}\right) , \end{aligned}$$

with

35 $$\begin{aligned} \tau _i=\dfrac{p_i\alpha _i}{\beta _i}, \quad A_i = \ln (m_i) - \ln (r_i) + p_i\left[ \ln (\beta _i+\mu _i)-\ln \beta _i\right] , \quad i=1,2, \end{aligned}$$

where $\alpha _i$ is defined in (33). The existence of (34) assumes that the boundary or semi-trivial equilibrium (29) exists or that condition (30) is true for $(i,j)=\{(1,2),(2,1)\}$. Again, as we did in (29), we can use l’Hôpital’s rule and the assumption that $p_i/\beta _i$ is constant, to find that $A\rightarrow (\mu _ip_i/\beta _i)\ln (m_i/r_i)$ in the limit where $p_i\rightarrow \infty$.

The trivial equilibrium and the semi-trivial equilibria are global asymptotic equilibria if the threshold condition for survival in Eq. (30) is not fulfilled for one or two species (see Lyapunov function in Appendix A).

**Fig. 4:**
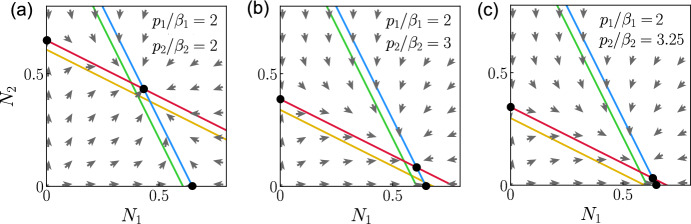
Phase plane with stable coexistence equilibrium. The nullclines obtained from (31)–(32) are shown in blue and in green for $N_1$ and in red and in yellow for $N_2$, when $p_1=p_2=1$ and when $p_1=p_2=20$ respectively, using $m_1=m_2=\mu _1 =\mu _2=0.2$, $a_1=a_2 =1$, $b_1=b_2=0.5$ and $r_1=r_2=1$, with the ratios $p_1/\beta _1=2$ and $p_2/\beta _2=2, 3$ and 3.25 in (**a**)–(**c**) respectively. The vector field (grey arrows) and the equilibrium points (black discs) are shown when $p_1=p_2=1$ (colour figure online)

We study the equilibrium point (34) in the cases of weak and strong interspecific competition. We are studying a projection of the solution on the plane $(N_1,N_2)$ from a phase space that represents the dynamical system from Eqs. (22)–(27). The plane $(N_1,N_2)$ is appropriate to study the solution around the equilibrium points as the variables $x_{i,k}$ and $y_{i,k}$ follow directly from the variables $N_i$, and as $f_{i}$ is simply the mean of $N_i$ during maturation. The variables $x_{i,k}$, $y_{i,k}$ and $f_{i}$ represent what is happening during maturation, while $N_i$ is the population at the current time. The equilibrium point in (34) exists for weak interspecific conditions if all the following conditions are true:

36 $$\begin{aligned} a_1 a_2 > b_1 b_2, \ \tau _2 a_2 A_1<\tau _1 b_2 A_2,\ \textrm{and} \ \tau _1 a_1 A_2 <\tau _2 b_1 A_1. \end{aligned}$$

The stable coexistence equilibrium when the interspecific competition is weak is shown in the phase planes of Fig. 4. The nullclines illustrated in blue for $N_1$ and in red for $N_2$, when $p_1=p_2=1$, are obtained from Eqs. (31) and (32) respectively. The intersection point of the nullclines in the positive quadrant corresponds to the stable coexistence equilibrium. Any solution starting at $N_1>0$ and $N_2>0$ will move towards the coexistence equilibrium when the conditions for its stability and existence are fulfilled. An example is shown when $p_1/\beta _1=p_2/\beta _2=2$ for $p_1=1$ in Fig. 4a where $\bar{N}_1=\bar{N}_2$, and when $p_1/\beta _1<p_2/\beta _2$ for $p_1=1$ in (b) and (c). $\bar{N}_1$ is being advantaged over $\bar{N}_2$ in (b) and (c). Figure 4 shows the nullclines in green for $N_1$ and in yellow for $N_2$ when $p_1=p_2=20$. A closer look at the green and yellow lines in (c) shows that the intersection of the two lines is outside the positive quadrant. We conclude that there exists $p_1=p_2\gg 1$ such that the species coexist for $p_1=p_2=1$ and such that species 1 excludes species 2 for $p_1=p_2\gg 1$, where the ratios $p_1/\beta _1$ and $p_2/\beta _2$ are maintained constant for $p_1=p_2=1$ and for $p_1=p_2=20$.

**Fig. 5:**
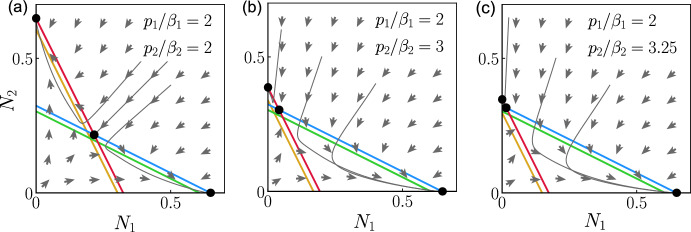
Phase plane with unstable coexistence equilibrium. The nullclines obtained from (31)–(32) are shown in blue and in green for $N_1$ and in red and in yellow for $N_2$, when $p_1=p_2=1$ and when $p_1=p_2=20$ respectively, using $m_1 = m_2 = \mu _1 = \mu _2 = 0.2$, $a_1 = a_2 = 1$, $b_1 = b_2= 2$ and $r_1=r_2=1$ with the ratios $p_1/\beta _1=2$ and $p_2/\beta _2=2, 3$ and 3.25 in (**a**)–(**c**) respectively. The vector field (grey arrows), the trajectories solutions (grey curves) and the equilibrium points (black discs) are shown for $p_1=p_2=1$ (colour figure online)

The equilibrium point in (34) exists for strong interspecific competition if all the following conditions are true:

37 $$\begin{aligned} a_1 a_2 \le b_1 b_2,\ \tau _2 a_2 A_1\ge \tau _1 b_2 A_2,\ \textrm{and} \ \tau _1 a_1 A_2\ge \tau _2 b_1 A_1. \end{aligned}$$

The conditions in (37) are obtained by reversing each inequality in (36). Figure  5 shows an example of bistability where the interspecific competition is strong and the coexistence equilibrium point is unstable. The asymptotic equilibrium depends on the starting point of the trajectory. The size of $\bar{N}_2$ in the equilibrium point $(\bar{N}_1=0,\bar{N}_2>0)$ is equal to that of $\bar{N}_1$ in the equilibrium point $(\bar{N}_1>0,\bar{N}_2=0)$, in Fig. 5a, as the unstable coexistence equilibrium is such that $\bar{N}_1=\bar{N}_2$. The size of $\bar{N}_2$ in the equilibrium point $(\bar{N}_1=0,\bar{N}_2>0)$ is smaller than $\bar{N}_1$ in the equilibrium point $(\bar{N}_1>0,\bar{N}_2=0)$, in Fig. 5b and c. The green and yellow lines in Fig. 5 representing the nullclines for $N_1$ and $N_2$ respectively show that the unstable coexistence equilibrium also exists in Fig. 5a–b when $p_1=p_2=20$. Figure 5c shows that there exists $p_1=p_2\gg 1$, with the ratios $p_1/\beta _1$ and $p_2/\beta _2$ maintained, such that the unstable coexistence equilibrium exists for $p_1=p_2=1$, where the winning species depends on the initial conditions, and such that the unstable coexistence equilibrium disappears for $p_1=p_2\gg 1$, where species 1 excludes species 2.

The strong interspecific competition conditions also include $a_1 a_2 = b_1 b_2$, where the coexistence equilibrium would exist only if the nullclines for $N_1$ and $N_2$ are superimposed, meaning that there is an infinity of equilibrium points.

#### Local stability

The system of Eqs. (14)–(15) is reduced to Eq. (14) for $i=1,2$ and linearised around equilibrium points $(\bar{N}_1,\bar{N}_2)$ from Sect. 3.2.1 where we substitute the exponential solution $[u(t) \ v(t)]^T=\exp {( \lambda t)} \ [b \ c]^T$. We use the properties of the Erlang function to determine $\int _{0}^{\infty }\exp {[\lambda (t-u)]}G(u\mid \beta ,p) \textrm{d}u=\exp {(\lambda t)}\left( \dfrac{\beta }{\beta +\lambda }\right) ^{p}$, as explained in Smith (2011). The characteristic equation can be obtained with the determinant of the following 2-by-2 matrix

38 $$\begin{aligned}&\begin{bmatrix} r_1\exp {\left( -\Delta _1 C_1\right) } h_1(\lambda ) - m_1 - \lambda &{}\quad 0\\ 0 &{}\quad r_2\exp {\left( -\Delta _2 C_2 \right) } h_2(\lambda ) - m_2 - \lambda \end{bmatrix} \nonumber \\&\quad -\begin{bmatrix} r_1a_1\Delta _1\exp {\left( -\Delta _1 C_1\right) } \,\dfrac{\textrm{d}f_1}{\textrm{d}u}\,\bar{N}_1&{} r_1 b_2\Delta _1 \exp {\left( -\Delta _1 C_1 \right) } \,\dfrac{\textrm{d}f_2}{\textrm{d}v}\,\bar{N}_1 \\ r_2b_1 \Delta _2\exp {\left( -\Delta _2 C_2 \right) } \,\dfrac{\textrm{d}f_1}{\textrm{d}u}\,\bar{N}_2&{} r_2a_2\Delta _2\exp {\left( -\Delta _2 C_2 \right) } \,\dfrac{\textrm{d}f_2}{\textrm{d}v}\, \bar{N}_2 \end{bmatrix} \end{aligned}$$

where $\dfrac{\textrm{d}f_1}{\textrm{d}u} =\dfrac{1-g_1(\lambda )}{\Delta _1\lambda }$, $\dfrac{\textrm{d}f_2}{\textrm{d}v} = \dfrac{1-g_2(\lambda )}{\Delta _2\lambda }$, $g_1(\lambda )=\left( \dfrac{\beta _1}{\beta _1+\lambda }\right) ^{p_1}$, $g_2(\lambda )=\left( \dfrac{\beta _2}{\beta _2+\lambda }\right) ^{p_2}$, $h_1(\lambda )=\left( \dfrac{\beta _1}{\beta _1+\lambda +\mu _1}\right) ^{p_1}$, $h_2(\lambda )=\left( \dfrac{\beta _2}{\beta _2+\lambda +\mu _2}\right) ^{p_2}$, $C_1 = a_1 \bar{f}_1 +b_2 \bar{f}_2$, $C_2 = a_2\bar{f}_2 +b_1 \bar{f}_1$, and where $\bar{f}_1$ and $\bar{f}_2$ can be expressed in terms of $\bar{N}_1$ and $\bar{N}_2$ respectively, such that $\bar{f}_1=\dfrac{\tau _1\bar{N}_1}{\Delta _1}$ and $\bar{f}_2=\dfrac{\tau _2\bar{N}_2}{\Delta _2}$, and assuming that $\lambda \ne 0$, $\lambda \ne -(\mu _i+\beta _i)$ and $\lambda \ne - \beta _i$.

The complete expression of the characteristic equation is given in Appendix B, Eq. (B16). Here, we give the specific characteristic equations around the trivial and semi-trivial equilibrium points and the corresponding stability conditions.

The eigenvalues when $(\bar{N}_1,\bar{N}_2)=(0,0)$ are obtained by solving the characteristic equation

39 $$\begin{aligned} \left[ \lambda + m_1 -r_1\left( \dfrac{\beta _1}{\beta _1 +\lambda +\mu _1}\right) ^{p_1}\right] \left[ \lambda + m_2-r_2 \left( \dfrac{\beta _2}{\beta _2+\lambda +\mu _2}\right) ^{p_2}\right] =0, \end{aligned}$$

The equilibrium point $(\bar{N}_1,\bar{N}_2)=(0,0)$ is stable if $\ln (m_1) - \ln (r_1) + p_1\left[ \ln (\beta _1+\mu _1) -\ln \beta _1\right] > 0$ and $\ln (m_2) - \ln (r_2) +p_2\left[ \ln (\beta _2+\mu _2)-\ln \beta _2\right] > 0$, corresponding respectively to $A_1 > 0$ and $A_2 > 0$, and the equilibrium point is unstable otherwise, meaning that if both species are not fit for survival, both species are extinct, and if both species are fit for survival, the solution will move away from the equilibrium $(\bar{N}_1,\bar{N}_2)=(0,0)$ to competitive exclusion or coexistence. The characteristic equation becomes

40 $$\begin{aligned}&\left\{ \lambda ^2+m_1\lambda -r_1\exp (a_1\tau _1\bar{N}_1) \left( \lambda \left( \dfrac{\beta _1}{\beta _1+\lambda +\mu _1}\right) ^{p_1} -a_1\bar{N}_1\left[ 1-\left( \dfrac{\beta _1}{\beta _1 +\lambda }\right) ^{p_1}\right] \right) \right\} \nonumber \\&\left[ \lambda +m_2 - r_2 \exp {\left( - b_1 \dfrac{\Delta _2\tau _1\bar{N}_1}{\Delta _1}\right) } \left( \dfrac{\beta _2}{\beta _2+\lambda +\mu _2}\right) ^{p_2}\right] = 0, \end{aligned}$$

when the equilibrium point is the competitive exclusion such that $N_1>0$ and $N_2=0$ and the characteristic equation becomes

41 $$\begin{aligned}&\left\{ \lambda ^2+m_2 \lambda -r_2 \exp (a_2\tau _2\bar{N}_2) \left( \lambda \left( \dfrac{\beta _2}{\beta _2+\lambda +\mu _2}\right) ^{p_2} -a_2\bar{N}_2\left[ 1-\left( \dfrac{\beta _2}{\beta _2 +\lambda }\right) ^{p_2}\right] \right) \right\} \nonumber \\&\left[ \lambda +m_1 -r_1 \exp {\left( -b_2 \dfrac{\Delta _1\tau _2 \bar{N}_2}{\Delta _2}\right) }\left( \dfrac{\beta _1}{\beta _1 +\lambda +\mu _1}\right) ^{p_1}\right] , \end{aligned}$$

when $$N_1=0$$ and $$N_2>0$$.

Equations (40) and (41) can be expressed as $M_{i,1}(\lambda )M_{i,2}(\lambda )=0$ where $M_{i,1}(\lambda )$ correspond to the first line of the right hand side of the equation and $M_{i,2}(\lambda )$ is the second line of the right hand side of the equation. $M_{i,1}(\lambda ) = 0$ have roots with negative real parts if the critical condition of survival (30) of the species *i* is fulfilled. $M_{i,2}(\lambda ) = 0$ in Eq. (40) have roots with negative real parts if $\tau _1 a_1 A_2\ge \tau _2 b_1 A_1$, and $M_{i,2} (\lambda ) = 0$ in Eq. (41) have roots with negative real parts if $\tau _2 a_2 A_1\ge \tau _1 b_2 A_2$. We conclude that the conditions of stability of existing equilibrium points $(\bar{N}_1,0)$ or $(0,\bar{N}_2)$ correspond partially to the conditions of existence (37) of the unstable coexistence equilibrium. At the opposite, the conditions of stability that make equilibrium points $(\bar{N}_1,0)$ or $(0,\bar{N}_2)$ unstable correspond partially to the conditions of existence (36) of the stable coexistence equilibrium.

We give an example of how to study local stability when $p_1=p_2=1$. Setting $p_1=p_2=1$ in (39) yields $[\lambda ^2+ 2\lambda (m_1+\beta _1+\mu _1) +m_1(\beta _1+\mu _1)-r_1\beta _1][\lambda ^2+ 2\lambda (m_2+\beta _2+\mu _2) + m_2(\beta _2+\mu _2)-r_2\beta _2] =0$ for $\lambda \ne \beta _1+\mu _1$. The eigenvalues are all real and negative if $m_1(\beta _1+\mu _1)>r_1\beta _1$ and $m_2(\beta _2+\mu _2)>r_2\beta _2$ equivalent to $A_1 > 0$ and $A_2 > 0$, where $A_i$ is defined in (35). The characteristic Eq. (40) when $p_1=p_2=1$ is in the form $M_{1,1}(\lambda ) M_{1,2}(\lambda )$, where $M_{1,1}(\lambda )$ is a polynomial of third order and $M_{1,2}(\lambda )$ is a polynomial of second order, that can be studied using the Routh-Hurwitz criterion for stability. The roots of polynomial $M_{1,1}(\lambda )$ all have negative real parts if the conditions in (28) are fulfilled, and the roots of $M_{1,2}(\lambda )$ all have negative real parts if $a_1 \alpha _1 \Delta _1 \left[ \ln (m_2) - \ln (r_2) + \ln (\beta _2+\mu _2) - \ln (\beta _2)\right] \ge b_1 \alpha _2 \Delta _2 \left[ \ln (m_1) - \ln (r_1) + \ln (\beta _1+\mu _1) - \ln (\beta _1)\right]$, corresponding to the condition $\tau _1 a_1 A_2\ge \tau _2 b_1 A_1$ from (37) when $\alpha _1$ and $\alpha _2$ approach one. The polynomial of order six corresponding to the characteristic equation for the coexistence equilibrium is given in Eq. (B17). The Routh-Hurwitz criterion can be verified with the help of symbolic software.

We now give the characteristic equations when $p_1\rightarrow \infty$ and $p_2\rightarrow \infty$ and when $p_1/\beta _1$ and $p_2/\beta _2$ are constant. We use l’Hôpital’s rule to determine that $g_1(\lambda )\rightarrow \exp {(-p_1\lambda /\beta _1)}$, $g_2(\lambda )\rightarrow \exp {(-p_2\lambda /\beta _2)}$, $h_1(\lambda )\rightarrow \exp {(-p_1(\lambda +\mu _1)/\beta _1)}$ and $h_2(\lambda )\rightarrow \exp {(-p_2(\lambda +\mu _2)/\beta _2)}$. The characteristic equation around $(\bar{N}_1,\bar{N}_2)=(0,0)$ is a transcendental equation that can be separated into two equations, such as $r_i\exp {(-p_i/\beta _i(\lambda +\mu _i))} - m_i - \lambda =0$, for $i=1,2$. Each transcendental equation could be rearranged in the form $z-c -d \exp {(-z))}=0$ if $z=p_i/\beta _i\lambda$, $c=-p_i/\beta _i m_i$ and $d=r_ip_i/\beta _i\exp {(-p_i/ \beta _i\mu _i)}$. We use the theorem that states that *z* has a negative real part if $c+d<0$ and $d\ge c$, as proved in Hayes (1950) and in Smith (2011). We find that the eigenvalues have a negative real part if $\ln (r_1/m_1)+p_1/\beta _1\mu _1> 0$ and $\ln (r_2/m_2)+p_2/\beta _2\mu _2> 0$, meaning that $(\bar{N}_1,\bar{N}_2)=(0,0)$ is stable if the critical conditions for survival for species 1 and 2 are not fulfilled and unstable otherwise. The characteristic equation around $(\bar{N}_i>0,\bar{N}_j=0)$ can be separated into two transcendental equations. The first transcendental equation is in the form $P(\lambda )+Q(\lambda )\exp {(-\beta _i/p_i\lambda )}$ where $P(\lambda )=\lambda ^2-m_i\lambda +r_ia_i\bar{N}_i\exp {(-\tau _i a_i \bar{N}_i)}$ and $Q(\lambda )=-r_i\exp {(-\tau _i a_i \bar{N}_i)}(a_i\bar{N}_i+\lambda \exp {(-\mu _i\beta _i/p_i})$. We verified that the three following conditions necessary for absolute stability (Brauer 1987; Smith 2011) are satisfied for every value of the delay $\beta _i/p_i$ when $N_i>0$ and $N_j=0$. The first condition is that the real part of the roots of $P(\lambda )$ must be positive or zero. The second condition is $|Q({\text {Im}}(\lambda ))|<|P({\text {Im}}(\lambda ))|$, for any positive ${\text {Im}}(\lambda )$. The third condition is $\lim \nolimits _{|\lambda |\rightarrow \infty , {\text {Re}}(\lambda )\ge 0}\ |Q(\lambda )|/ |P(\lambda )|=0$. The second transcendental equation is in the form $z-c-d\exp {(-z)}=0$, where $z=\beta _j/p_j\lambda$, $c=-m_j\beta _j/p_j$ and $d=r_j \beta _j/p_j\exp {\{(b_i\tau _j)/ (a_i\tau _i)[\ln {(m_i/r_i)}+\beta _i/p_i\mu _i]-\beta _j/p_j\mu _j\}}$ such that *z* has negative real part if $\exp {\{(b_i\tau _j)/(a_i \tau _i)[\ln {(m_i/r_i)}+\beta _i/p_i\mu _i]-\beta _j/p_j\mu _j\}}<m_j/r_j$. The characteristic equation around the coexistence equilibrium point takes a more complicated form (see Appendix B). Such forms of transcendental equations with two discrete delays are approached in An et al. (2019). We remind the reader that the competition between two species with a discrete delay is also studied in Lin et al. (2022).

### Distributed delay promotes coexistence and survival

It is useful to understand how the equilibrium for a species moves from extinction to persistence depending on the parameters of the model. We use the following method to determine the values of $\bar{N}_1$ and $\bar{N}_2$ depending on the parameters of the model. We determine if the species *i* can survive by verifying if condition (30) is true. The species that does not pass the test in (30), independently of competition, is extinct and the species that does pass the test survives if the other is extinct. If condition (30) is true for both species, we establish the existence of the coexistence equilibrium point (34), then we determine if the coexistence equilibrium is stable (36) or unstable (37). It is possible that the coexistence equilibrium does not exist in the positive quadrant. We evaluate the intersection of the nullclines (31) and (32) to determine the outcome of competitive exclusion. An intersection in the quadrant where $\bar{N}_1$ is positive and $\bar{N}_2$ is negative means that species 1 excludes species 2 if $a_1a_2<b_1b_2$, and that species 2 excludes species 1 otherwise. An intersection in the quadrant where $\bar{N}_1$ is negative and $\bar{N}_2$ is positive means that species 2 excludes species 1 if $a_1a_2<b_1b_2$, and that species 1 excludes species 2 otherwise.

**Fig. 6:**
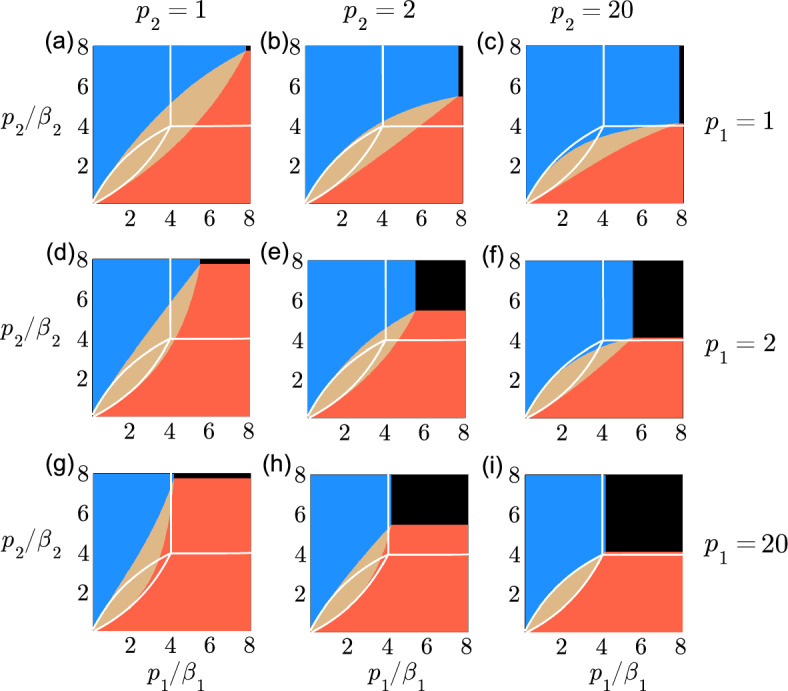
Extinction, survival and coexistence as a function of $p_1$ and $p_2$. The region of competitive exclusion is represented in blue if $\bar{N}_1>0$ and in red if $\bar{N}_2>0$. The region of coexistence where $\bar{N}_1>0$ and $\bar{N}_2>0$ is represented in brown. The region where both populations are extinct is represented in black. The conditions for existence and the stability of each region are obtained with $\mu _1 = \mu _2 = m_1 = m_2 = 0.3$, $a_1 = a_2 = 1$, $b_1 = b_2 = 0.5$, $r_1=r_2=1$, $p_1=1$ in (**a**)–(**c**), $p_1=2$ in (**d**)–(**f**), $p_1=20$ in (**g**)–(**i**), $p_2=1$ in (**a**), (**d**) and (**g**), $p_2=2$ in (**b**), (**e**) and (**h**), and $p_2=20$ in (**c**), (**f**) and (**i**). The axis representing $p_1/\beta _1$ and $p_2/\beta _2$ are shown from 0.1 to 8. The white curve corresponds to the frontiers of each region in the limit where $p_1\rightarrow \infty$ and $p_2\rightarrow \infty$, equivalent to the kernel $\delta (s_i-p_i/\beta _i)$ of a discrete delay (colour figure online)

Figure 6 gives examples of how coexistence, competitive exclusion and extinction depend on the shape $p_i$. Figure 6 is subdivided in 9 different cases where $p_1=1,2$ and 20, and $p_2=1, 2$ and 20. Each case shows the region of coexistence in brown, the region where species 1 wins in blue, the region where species 2 wins in red and the region where both species are extinct in black. The regions are illustrated on a map where $p_1/\beta _1$ is on the horizontal coordinate and where $p_2/\beta _2$ is on the vertical coordinate. Using the functional form $p_i/\beta _i$ for our unfolding parameters allows us to compare the results of the model with a distributed delay to the model with a discrete delay, as $p_i/\beta _i$ represents the maturation duration in the discrete delay kernel when $p_i\rightarrow \infty$.

As $p_1/\beta _1$ and $p_2/\beta _2$ both vary from 1/10 to 8, as a result $\beta _1$ and $\beta _2$ vary from 10 to 1/8 in Fig. 6a, and both species are extinct in the region where $\beta _1<1/7.8$ and $\beta _2<1/7.8$, or where $p_1/\beta _1 > 7.8$ and $p_2/\beta _2 > 7.8$. The region of coexistence shown in brown in Fig. 6a is approximately four times larger than the region of coexistence delimited by the white curve representing the region of coexistence for the discrete delay. In Fig. 6i, where $\beta _1$ and $\beta _2$ vary from 200 to 20/8, both species are extinct in the region where $p_1/\beta _1>4$ and $p_2/\beta _2>4$, or where $\beta _1<5$ and $\beta _2<5$. The region of coexistence shown in brown in Fig. 6i approximates the corresponding region delimited by the white curve for a discrete delay. The species with the shortest maturation is advantaged when a species excludes another species in Fig. 6. The parameter $p_i$ plays a role in the persistence of the species, as it corresponds to the number of events expected to occur at fixed rate of maturation. Species 1, for example, is advantaged at the expense of species 2 in Fig. 6b where $p_1=1$ and $p_2=2$.

We did not present the equivalent of Fig. 6 for strong interspecific competition. The regions of competitive exclusion (red or blue) and total extinction (black) shown in Fig. 6 for $a_1=a_2=1$ and $b_1=b_2=0.5$ would have the same shapes and areas if $b_1=b_2=2$. The region of coexistence in brown would become a region of bistability.

**Fig. 7:**
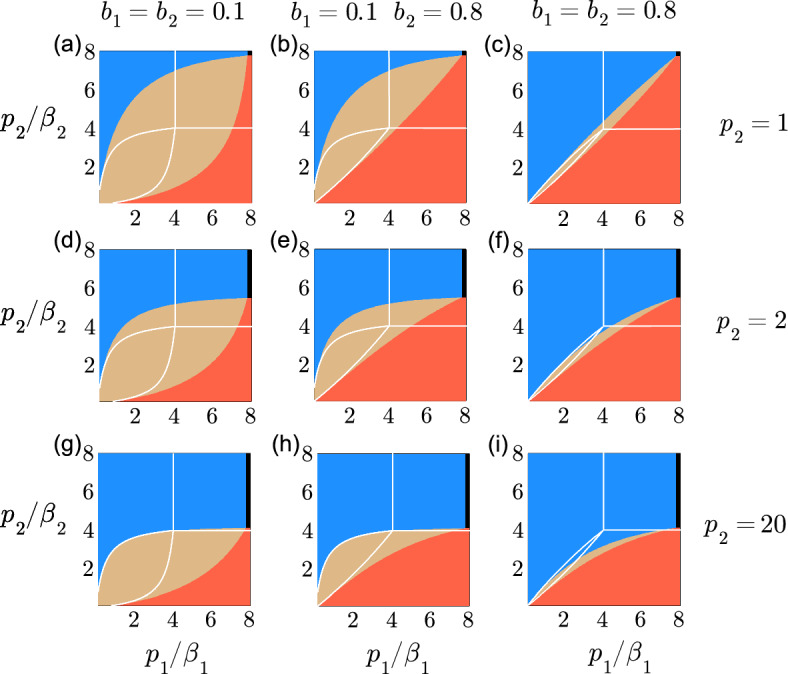
Extinction, survival and coexistence as a function of $p_2$, $b_1$ and $b_2$. The region of competitive exclusion is represented in blue when $\bar{N}_1>0$ and in red when $\bar{N}_2>0$. The region of coexistence where $\bar{N}_1>0$ and $\bar{N}_2>0$ is represented in brown. The region where all populations are extinct is represented in black. The conditions for the existence and the stability of each region are obtained with $\mu _1 = \mu _2 = m_1 = m_2 = 0.3$, $a_1 = a_2 = 1$, $r_1=r_2=1$, $p_1=1$, $b_1=b_2=0.1$ in (**a**), (**d**) and (**g**), $b_1=0.1$ and $b_2=0.8$ in (**b**), (**e**) and (**h**), $b_1=b_2=0.8$ in (**c**), (**f**) and (**i**), $p_2=1$ in (**a**)–(**c**), $p_2=2$ in (**d**)–(**f**), and $p_2=20$ in (**g**)–(**i**). The axis representing $p_1/\beta _1$ and $p_2/\beta _2$ are shown from 0.1 to 8. The white curve corresponds to the frontiers of each region in the limit where $p_1\rightarrow \infty$ and $p_2\rightarrow \infty$, equivalent to the kernel $\delta (s_i-p_i/\beta _i)$ of a discrete delay (colour figure online)

The effect of species 2 on species 1, represented by the parameter $b_1$, and the effect of species 1 on species 2, represented by the parameter $b_2$, play a role in persistence and extinction, as shown for $a_1a_2<b_1b_2$ in Fig. 7, where $p_1=1$. The region of stable coexistence, coloured in brown, is larger when $b_1=b_2=0.1$ in Fig. 7a whereas the region is much smaller when $b_1=b_2=0.8$ in Fig. 7c. Species 2, shown in red, is advantaged at the expense of species 1 where $b_1=0.1$ and $b_2=0.8$ in Fig. 7c. Species 1 is advantaged over species 2 where $p_2=20$ in (g)–(i) when compared to the results in (a)–(c) where $p_2=1$, as the rates $\beta _2$ used in (g)–(i) are much smaller than the rates used in (a)–(c).

We can also interpret the results in Fig. 7 by comparing the regions of coexistence (brown) and survival or competitive exclusion (red and blue) to the regions delimited by the white curves representing the discrete delay. All the cases show that the distributed delay promotes coexistence of both species and the survival of a species. The region of total extinction for the discrete delay, delimited by the white lines, would be larger than the narrow black rectangle that represents the region of extinction for the distributed delay. The regions in Fig. 7 would have the same configurations for strong interspecific competition when $a_1=a_2=1$ if $b_1=b_2=10$ in (a), (d) and (g), $b_1=10$ and $b_2=1.25$ in (b), (e) and (h), and $b_1=b_2=1.25$ in (c), (f) and (i). The region in brown would represent the bistable equilibrium of strong interspecific competition. Figures 6 and 7 highlight the flexibility offered by the parameters of the distributed delay allowing for more coexistence and survival compared to the discrete delay. The results are shown for a restricted set of parameters in Figs. 6 and 7. Our analysis and an extensive exploration of the results for a larger domain of parameters show that the following observation remains true: the threshold for survival depending partially on the expression $\left[ (\beta _i+\mu _i)/\beta _i\right] ^{p_i}$ is always smaller for $p_i=1$ than for $p_i\rightarrow \infty$ if $p_i/\beta _i$ remains constant.

### Biological example

We give an applied example of the model to show possible outcomes with real biological parameters. Two species of mosquitoes larvae, *Anopheles gambiae* sensu stricto and *Anopheles arabiensis*, vectors of malaria in Africa, can be found in the same reservoirs of water where they mature. We use assumptions about the biology of both species from the literature (Kirby and Lindsay 2009; Paaijmans et al. 2009). Species 1, *An. gambiae* s.s., has a shorter maturation than species 2, *An. arabiensis*. Species 1 has also the biggest carrying capacity, as the female of species 1 reaches a smaller size than the female of species 2 at full growth. The maturation delay could be shortened for both species, in warmer water ponds for example (Kirby and Lindsay 2009; Agyekum et al. 2022).

**Fig. 8:**
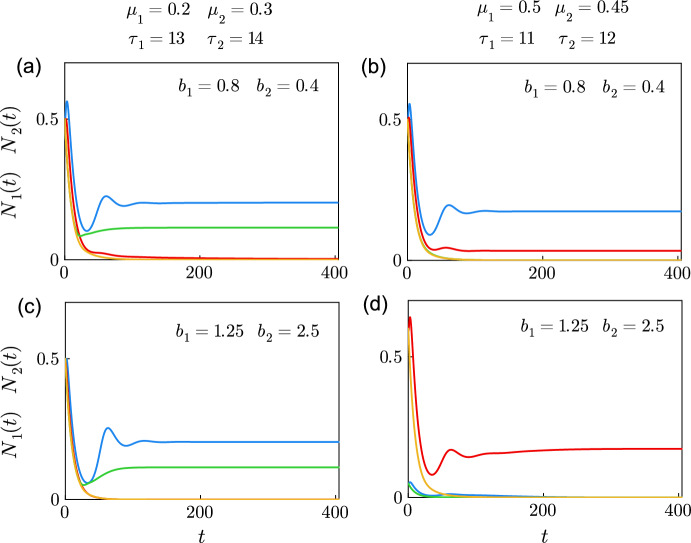
Coexistence and competitive exclusion between species of mosquitoes. Numerical solutions of the system of ODE (22)–(27) from initial conditions $N_1(0)=N_2(0)=0.5$ in (**a**)–(**c**), $N_1(0)=0.05$ and $N_2(0)=0.6$ in (**d**), with $x_{i,k}(0)=y_{i,k}(0)=f_{i}(0)=0$, where $r_1 = r_2 =5$ eggs per individual per day, $m_1=m_2=0.1$, $a_1 = a_2 =1$, $b_1=0.8$, $b_2=0.4$ in (**a**) and (**b**), $b_1=1.25$, $b_2=2.5$ in (**c**) and (**d**), $\mu _1=0.2$/day, $\mu _2=0.3$/day, $\tau _1=13$ days and $\tau _2=14$ days in (**a**) and (**c**), $\mu _1=0.5$/day, $\mu _2=0.45$/day, $\tau _1=11$ days and $\tau _2=12$ days in (**b**) and (**d**). $N_1(t)$, representing the *Anopheles gambiae* sensu stricto population, is shown in blue when $p_1=p_2=1$ and in green when $p_1=p_2=20$. $N_2(t)$, representing the *Anopheles arabiensis* population, is shown in red when $p_1=p_2=1$ and in yellow when $p_1=p_2=20$ (colour figure online)

Figure 8 shows the populations of *An. gambiae* s.s. ($N_1(t)$) and *A. arabiensis* ($N_2(t)$), where the non dimensional $N_i(t)=1$ corresponds to $\hat{N}_i(\hat{t})=\hat{K}_i$. The maturation in Fig. 8 is delayed and the distribution of individual delays within the population of one species is heterogeneous, at opposed to the classic Lotka–Volterra model where the maturation is instantaneous and homogeneous. We selected maturation delays and mortality rates from Kirby and Lindsay (2009) that are physically plausible for both species. The parameters $\beta _1$ and $\beta _2$ in Fig. 8 are chosen so that $\tau _1=13$ and $\tau _2=14$ in (a), $\tau _1=11$ and $\tau _2=12$ in (b). The shifts corresponding to the truncation are chosen so that $\alpha _1=\alpha _2=0.99$: this gives shifts $\Delta _1=59.9$ and $\Delta _2=64.5$ in (a) for $p_1=p_2=1$, and $\Delta _1=20.7$ and $\Delta _2=22.3$ in (a) for $p_1=p_2=20$.

The potential for coexistence that arises from weak interspecific competition is illustrated in Fig. 8a, b when $p_1 = p_2 = 1$: for longer delays in (a), and for shorter delays but higher mortality rates in (b). Coexistence fails in both cases when $p_1 = p_2 = 20$: in (a), *A. arabiensis* becomes extinct, and in (b) both species reach extinction. Coexistence of *An. gambiae* (blue) and *A. arabiensis* (red) is possible in Fig. 8b when $p_1=p_2=1$.

The solutions in Fig. 8c and d are obtained with the same parameters as in (a) and (b) respectively, except for $a_1$, $a_2$, $b_1$ and $b_2$ that we modified to satisfy the condition for strong interspecific competition. *An. gambiae* s.s. excludes *A. arabiensis* in Fig. 8c. *A. arabiensis* wins the competition with appropriate initial conditions in Fig. 8d only when $p_1=p_2=1$.

Transient oscillations can be observed following $t=0$ in Fig. 8a–d. Preliminary results seem to indicate that the peak of the transient oscillations varies with $r_i$. Further study is required to understand the relationship between the transient oscillations and the parameters of the model.

## Conclusion

We generalised the alternative delayed Lotka–Volterra model by reformulating the model with a distributed delay. The kernel of the Erlang distribution was used for a more realistic representation of the heterogeneity found in the length of maturation in a species. The distributed delay separated the mature from the immature individuals, to represent a species where competition is more important for immature individuals and where maturation time is long compared to lifetime. We used the linear chain trick to approximate the DDEs of the model by a system of ODEs. We showed how the survival of a species depends on the rate of maturation being able to compensate for the rate of loss due to mortality of adults and immature individuals. A species fit for survival enters into competition with another species, leading to competitive exclusion, to coexistence of both species, or to a bistable equilibrium. The necessary conditions for stability were shown for the equilibrium points in function of the parameters of the model. We also gave sufficient conditions to determine a global asymptotic equilibrium. We highlighted differences of the model with a distributed delay as compared to the discrete delay. The model has the overall effect of promoting coexistence and survival of the species. The system is able to induce stable coexistence for a skewed distribution of the maturation delay, whereas a competitive exclusion would be expected for a discrete maturation delay, such that a model that inaccurately assumes a discrete delay would give a completely incorrect result. Another difference of the model is that the chances for species with less fitness to “win” in competition are increased in the bistable equilibrium for a skewed distribution, whereas the extinction of the species would be expected for a discrete maturation delay, as showed in the biological example in Sect. 3.4. The model could be extended for three or more species, so we could study whether the model property of allowing more coexistence is maintained in higher dimensions. Future work would also require study of the transient oscillations to see how well they describe the transient behaviour of a population at equilibrium after a perturbation is introduced.

## Data Availability

The Julia code is available on GitHub. An implementation in R is also available in the same repository.

## Appendix A: Lyapunov function

Assume we have an ODE system

A1 $$\begin{aligned} \dot{\textbf{x}} = h(\textbf{x}) \end{aligned}$$

with an equilibrium at $\textbf{x} = 0$ for vector-valued $\textbf{x}$.

Then if we can define a *Lyapunov function* $V(\textbf{x})$ such that:

A2 $$\begin{aligned} V(\textbf{x})&= 0 \text { iff }\textbf{x} = 0, \end{aligned}$$

A3 $$\begin{aligned} V(\textbf{x})&> 0\text { iff }\textbf{x} \ne 0, \text {and} \end{aligned}$$

A4 $$\begin{aligned} \dot{V}(\textbf{x}) = \frac{d}{dt} V(\textbf{x}) = \sum _{i=1}^n \frac{\partial V}{\partial x_i} h_i(\textbf{x})&= \nabla V \cdot h(\textbf{x}) < 0\text { for all }x \ne 0 \end{aligned}$$

then the equilibrium is globally asymptotically stable.

It is possible to show that there is a global asymptotic equilibrium that does not depend on the initial conditions. As mentioned in Sect. 3.2, the existence of an equilibrium in Eqs. (22)–(27) is equivalent to one in the condensed set Eqs. (22)–(24). We thus use this condensed form of Eqs. (22)–(27), where $f_i = \int _{t-\int {\hat{G}\left( u\mid \beta ,p,\Delta _i\right) \textrm{d}u}}^{t} N_i(s) \textrm{d}s \ge 0$ and thus $\exp (-f_i) < 1$, and where no differential equations are attributed to $\textrm{d}f_i/\textrm{d}t$ and $y_{i,k}$. We first set

$$\begin{aligned} \textbf{x} = \left( N_1 - \bar{N}_1, x_{1,1} - \bar{N}_1, \ldots , x_{1,p_1} - \bar{N}_1, N_2 - \bar{N}_2, x_{2,1} - \bar{N}_2, \ldots , x_{2,p_2} - \bar{N}_2 \right) , \end{aligned}$$

where $\bar{N}_i$ are the values at a given equilibrium point as defined at the start of Sect. 3.2.1, to satisfy Eq. (A2) above. Then if we set $D_i = \displaystyle \frac{\mu _i + \beta _i}{1 - \exp \left( -\beta _i \Delta _i \right) e_{p_i - 1} \left( \beta _i \Delta _i \right) }$ and define the matrices

A5 $$\begin{aligned} \mathbf {A'}_i = \begin{bmatrix} -m_i/r_i &{}\quad 0 &{}\quad 0 &{}\quad 0 \\ D_i &{}\quad -D_i &{}\quad 0 &{}\quad 0 \\ 0 &{}\quad \ddots &{} \quad \ddots &{}\quad 0 \\ 0 &{}\quad 0 &{}\quad D_i &{} \quad -D_i \end{bmatrix} \end{aligned}$$

and

A6 $$\begin{aligned} \textbf{A} = \begin{bmatrix} \mathbf {A'}_1 &{}\quad \textbf{0} \\ \textbf{0} &{}\quad \mathbf {A'}_2 \end{bmatrix}, \end{aligned}$$

we can describe our model as

A7 $$\begin{aligned} \dot{\textbf{x}} = \textbf{A} \textbf{x} + g(\textbf{x}) \end{aligned}$$

where $g(\textbf{x})$ is a nonlinear function with equilibrium also at $\textbf{x}=0$, defined by

A8 $$\begin{aligned} g(\textbf{x}) = \begin{bmatrix} \dfrac{\beta _1}{\beta _1+\mu _1}x_{1,p_1} \exp \left[ -\Delta _1 \left( \dfrac{a_1 f_1}{\Delta _1} +\dfrac{b_2 f_2}{\Delta _2}\right) \right] \\ 0 \\ \vdots \\ 0\\ \dfrac{\beta _2}{\beta _2+\mu _2} x_{2,p_2} \exp \left[ -\Delta _2 \left( \dfrac{a_2 f_2}{\Delta _2} +\dfrac{b_1 f_1}{\Delta _1}\right) \right] \\ 0 \\ \vdots \\ 0\\ \end{bmatrix}. \end{aligned}$$

Then if we define our Lyapunov function *V* as

A9 $$\begin{aligned} V(\textbf{x}) = \textbf{x}^{T} \, \textbf{B} \, \textbf{x} \end{aligned}$$

given a positive-definite matrix $\textbf{B}$, then Eq. (A3) above is satisfied by definition. As $\textbf{A}$ is a lower bidiagonal matrix, its eigenvalues $\varvec{\lambda }$ are equal to its diagonal values, i.e.

A10 $$\begin{aligned} \varvec{\lambda } = \left[ -m_1 / r_1, -D_1, \ldots , -D_1, -m_2/r_2, -D_2, \ldots , -D_2 \right] \quad . \end{aligned}$$

We then select $\textbf{B}=-1/(2\varvec{\lambda })\, \textbf{I}$, which will by definition have positive eigenvalues and thus be positive-definite as required.

We must now show that

A11 $$\begin{aligned} \dot{V}(\textbf{x}) = \textbf{x}^{T} \left( \textbf{A}^{T} \textbf{B} + \textbf{B} \textbf{A} \right) \textbf{x}+ 2 \textbf{x}^{T}\, \textbf{B}\, g(\textbf{x}) \end{aligned}$$

is negative everywhere corresponding to the remaining condition, Eq. (A4). The matrix $\textbf{C} = \textbf{A}^{T} \textbf{B} + \textbf{B} \textbf{A}$ has the form

A12 $$\begin{aligned} \textbf{C} = \begin{bmatrix} \mathbf {C'} &{} \textbf{0} \\ \textbf{0} &{} \mathbf {C'} \end{bmatrix}, \end{aligned}$$

where

A13 $$\begin{aligned} \mathbf {C'} = \begin{bmatrix} -1 &{}\quad 1/2 &{}\quad 0 &{}\quad 0 \\ 1/2 &{}\quad -1 &{}\quad \ddots &{}\quad 0 \\ 0 &{}\quad \ddots &{}\quad \ddots &{}\quad 1/2 \\ 0 &{}\quad 0 &{}\quad 1/2 &{}\quad -1 \end{bmatrix} \end{aligned}$$

and is negative-definite as required (See the Mathematica file on GitHub).

The full equation for $\dot{V}$ is thus:

A14 $$\begin{aligned} \dot{V}(\textbf{x})&= -\left[ \left( N_1 - \bar{N}_1\right) ^2 +\sum _{k=1}^{p_1}\left( x_{1,k} - \bar{N}_1\right) ^2 +\left( N_2 - \bar{N}_2\right) ^2 + \sum _{k=1}^{p_2} \left( x_{2,k} - \bar{N}_2\right) ^2 \right] \nonumber \\&\quad + \left( N_1 - \bar{N}_1\right) \left( x_{1,1} - \bar{N}_1\right) +\left( N_2 - \bar{N}_2\right) \left( x_{2,1} - \bar{N}_2\right) \nonumber \\&\quad + \sum _{k=1}^{p_1-1}\left( x_{1,k} - \bar{N}_1\right) \left( x_{1,k+1} - \bar{N}_1\right) + \sum _{k=1}^{p_2-1} \left( x_{2,k} - \bar{N}_2\right) \left( x_{2,k+1} - \bar{N}_2\right) \nonumber \\&\quad +\left( N_1 - \bar{N}_1\right) \left( x_{1,p_1} - \bar{N}_1\right) \dfrac{r_1}{m_1}\left( \dfrac{\beta _1}{\beta _1+\mu _1}\right) ^{p_1} \exp \left[ -\Delta _1 \left( \dfrac{a_1f_1}{\Delta _1} +\dfrac{b_2f_2}{\Delta _2}\right) \right] \nonumber \\&\quad +\left( N_2 - \bar{N}_2\right) \left( x_{2,p_2} - \bar{N}_2\right) \dfrac{r_2}{m_2}\left( \dfrac{\beta _2}{\beta _2+\mu _2}\right) ^{p_2} \exp \left[ -\Delta _2 \left( \dfrac{a_2f_2}{\Delta _2} +\dfrac{b_1f_1}{\Delta _1}\right) \right] , \end{aligned}$$

For a periodic sequence of real numbers $\{ c_1, \ldots , c_n, \ldots \}$ where $c_{n+1} \equiv c_1$,

A15 $$\begin{aligned} \sum _{i=1}^n (c_i - c_{i+1})^2&> 0 \nonumber \\ \therefore \qquad \sum _{i=1}^n \left( c_i^2 + c_{i+1}^2 - 2 c_i c_{i+1} \right)&> 0 \nonumber \\ \therefore \qquad \sum _{i=1}^n c_i^2&> \sum _{i=1}^n c_i c_{i+1}, \end{aligned}$$

so using Eq. A15 with the sequences $\{ N_i - \bar{N}_i, x_{i,k} - \bar{N}_i, \ldots , x_{i,p_i} - \bar{N}_i, \ldots \}$ for $i = 1, 2$ gives $\dot{V}(\textbf{x}) < 0$ as required if

$$\begin{aligned} \dfrac{r_1}{m_1}\left( \dfrac{\beta _1}{\beta _1+\mu _1}\right) ^{p_1} \exp \left[ -\Delta _1 \left( \dfrac{a_1f_1}{\Delta _1} +\dfrac{b_2f_2}{\Delta _2}\right) \right]&< 1 \quad \text {and} \\ \dfrac{r_2}{m_2}\left( \dfrac{\beta _2}{\beta _2+\mu _2}\right) ^{p_2} \exp \left[ -\Delta _2 \left( \dfrac{a_2f_2}{\Delta _2} +\dfrac{b_1f_1}{\Delta _1}\right) \right]&< 1, \end{aligned}$$

and given our previous statement that $f_i \ge 0$, this reduces to $\dfrac{r_i}{m_i}\left( \dfrac{\beta _i}{\beta _i+\mu _i}\right) ^{p_i} < 1$ for $i = 1, 2$. $\square$

## Appendix B: Characteristic equations

The characteristic equation obtained from the determinant of (38) is

B16 $$\begin{aligned}&\left\{ \lambda ^2+m_1\lambda -r_1 \exp {\left[ - \left( a_1 \tau _1\bar{N}_1 +b_2 \dfrac{\Delta _1\tau _2\bar{N}_2}{\Delta _2}\right) \right] }\right. \nonumber \\&\qquad \left. \,\left( \lambda \left( \dfrac{\beta _1}{\beta _1 +\lambda +\mu _1}\right) ^{p_1} -a_1\bar{N}_1 \left[ 1-\left( \dfrac{\beta _1}{\beta _1+\lambda } \right) ^{p_1}\right] \right) \right\} \nonumber \\&\qquad \left\{ \lambda ^2+m_2 \lambda - r_2 \exp {\left[ - \left( a_2\tau _2\bar{N}_2 +b_1 \dfrac{\Delta _2\tau _1\bar{N}_1}{\Delta _1}\right) \right] }\right. \nonumber \\&\qquad \left. \,\left( \lambda \left( \dfrac{\beta _2}{\beta _2 +\lambda +\mu _2}\right) ^{p_2} -a_2\bar{N}_2 \left[ 1-\left( \dfrac{\beta _2}{\beta _2+\lambda } \right) ^{p_2}\right] \right) \right\} \nonumber \\&\quad -r_1r_2b_1 b_2 \bar{N}_1 \bar{N}_2 \nonumber \\&\exp {\left[ - \left( a_1 \tau _1\bar{N}_1 +b_2 \dfrac{\Delta _1\tau _2\bar{N}_2}{\Delta _2} +a_2\tau _2 \bar{N}_2 +b_1 \dfrac{\Delta _2\tau _1 \bar{N}_1}{\Delta _1}\right) \right] }\nonumber \\&\quad \left[ 1-\left( \dfrac{\beta _1}{\beta _1+\lambda } \right) ^{p_1}\right] \left[ 1-\left( \dfrac{\beta _2}{\beta _2 +\lambda }\right) ^{p_2}\right] = 0 \end{aligned}$$

after substituting the equilibrium point $(\bar{N}_1,\bar{N}_2)$.

Studying the local stability around the coexistence equilibrium point in (34) implies solving the characteristic equation

B17 $$\begin{aligned}&\left\{ \lambda ^2+m_1\lambda -m_1\left( \dfrac{\beta _1+\mu _1}{\beta _1}\right) ^{p_1}\right. \nonumber \\&\qquad \left. \left( \lambda \left( \dfrac{\beta _1}{\beta _1 +\lambda +\mu _1}\right) ^{p_1} -a_1\bar{N}_1 \left[ 1-\left( \dfrac{\beta _1}{\beta _1+\lambda } \right) ^{p_1}\right] \right) \right\} \nonumber \\&\left\{ \lambda ^2+m_2 \lambda -m_2 \left( \dfrac{\beta _2+\mu _2}{\beta _2}\right) ^{p_2}\right. \nonumber \\&\qquad \left. \left( \lambda \left( \dfrac{\beta _2}{\beta _2 +\lambda +\mu _2}\right) ^{p_2} -a_2\bar{N}_2 \left[ 1-\left( \dfrac{\beta _2}{\beta _2+\lambda } \right) ^{p_2}\right] \right) \right\} \nonumber \\&\quad -b_1 b_2 m_1 m_2 \bar{N}_1 \bar{N}_2 \left( \dfrac{\beta _1+\mu _1}{\beta _1}\right) ^{p_1} \left( \dfrac{\beta _2+\mu _2}{\beta _2}\right) ^{p_2} \nonumber \\&\quad \left[ 1-\left( \dfrac{\beta _1}{\beta _1+\lambda } \right) ^{p_1}\right] \left[ 1-\left( \dfrac{\beta _2}{\beta _2+\lambda }\right) ^{p_2}\right] = 0. \end{aligned}$$

The characteristic Eq. (B17) for the coexistence equilibrium $(\bar{N}_1,\bar{N}_2)$ when $p_1=1$ and $p_2=1$ is

B18 $$\begin{aligned}&\left\{ \beta _1(\lambda +m_1)(\beta _1+\lambda ) (\beta _1+\lambda +\mu _1)\right. \nonumber \\&\quad \left. -m_1\left( \beta _1+\mu _1 \right) \left[ \beta _1(\beta _1+\lambda ) -a_1\bar{N}_1 (\beta _1+\lambda +\mu _1) \right] \right\} \nonumber \\&\left\{ \beta _2(\lambda +m_2)(\beta _2+\lambda ) (\beta _2+\lambda +\mu _2)\right. \nonumber \\&\quad \left. -m_2 \left( \beta _2+\mu _2 \right) \left[ \beta _2(\beta _2+\lambda ) -a_2\bar{N}_2 (\beta _2+\lambda +\mu _2) \right] \right\} \nonumber \\&\quad -b_1 b_2 m_1 m_2 \bar{N}_1 \bar{N}_2 \left( \beta _1+\mu _1 \right) \left( \beta _2+\mu _2 \right) \nonumber \\&(\beta _1+\lambda )(\beta _2+\lambda )(\beta _1+\lambda +\mu _1) (\beta _2+\lambda +\mu _2)= 0. \end{aligned}$$

The characteristic equation for the coexistence equilibrium $(\bar{N}_1,\bar{N}_2)$ when $p_1\rightarrow \infty$ and $p_2\rightarrow \infty$ is

B19 $$\begin{aligned}&\left\{ \lambda ^2+m_1\lambda -\left[ m_1\lambda +m_1a_1\bar{N}_1 \exp {\left( \dfrac{p_1\mu _1}{\beta _1} \right) }\right] \exp {\left( -\dfrac{\mu _1 \lambda }{\beta _1}\right) } \right. \nonumber \\&\qquad \left. + m_1a_1\bar{N}_1 \exp {\left( \dfrac{p_1\mu _1}{\beta _1}\right) } \right\} \nonumber \\&\left\{ \lambda ^2+m_2\lambda -\left[ m_2\lambda +m_2a_2\bar{N}_2 \exp {\left( \dfrac{p_2\mu _2}{\beta _2} \right) }\right] \exp {\left( -\dfrac{\mu _2 \lambda }{\beta _2}\right) } \right. \nonumber \\&\qquad \left. + m_2a_2\bar{N}_2 \exp {\left( \dfrac{p_2\mu _2}{\beta _2}\right) } \right\} \nonumber \\&\quad -b_1 b_2 m_1 m_2 \bar{N}_1 \bar{N}_2 \exp {\left( \dfrac{\mu _1 p_1}{\beta _1}\right) } \exp {\left( \dfrac{\mu _2 p_2}{\beta _2}\right) } \nonumber \\&\quad \left[ 1- \exp {\left( -\dfrac{p_1}{\beta _1}\lambda \right) }\right] \left[ 1- \exp {\left( -\dfrac{p_2}{\beta _2}\lambda \right) }\right] = 0. \end{aligned}$$

## Appendix C: Numerical explorations

We solved Eqs. (22)–(27) for a large set of parameters to verify that no solutions contain limit cycles or strange attractors around the equilibrium points, and to confirm the results from Sect. 3.2. We set $\alpha =0.99$, $a_1=a_2=1$, $r_1=r_2=1$, and we varied $\beta _1$ from 0.25 to 20, $\beta _2$ from 0.25 to 20, $b_1$ from 0.25 to 10, $b_2$ from 0.25 to 10, $\mu$ from 0.25 to 30 and *m* from 1 to 10, altogether for $p_1=1$ and 20 and $p_2=1$ and 20. The parameters $r_1$, $r_2$ in the equations could be re-dimensionalised in terms of $m_1/r_1$ and $m_2/r_2$. The parameters $a_1$ and $a_2$ were fixed to one as what matters in determining the stability of the coexistence equilibrium is the ratio $a_1a_2/b_1b_2$.
